# Pharmacokinetics, safety, and antitumor activity of talazoparib monotherapy in Chinese patients with advanced solid tumors

**DOI:** 10.1007/s10637-023-01351-w

**Published:** 2023-05-12

**Authors:** Yang Luo, Ying Cheng, Chunjiao Wu, Hui Ye, Naihan Chen, Fan Zhang, Hua Wei, Binghe Xu

**Affiliations:** 1 National Cancer Center/Cancer Hospital, Chinese Academy of Medical Sciences and Peking Union Medical College, Beijing, China; 2 Department of Medical Oncology, Jilin Cancer Hospital, Changchun, Jilin, China; 3 Clinical Development, Development China, Pfizer Pharmaceutical Ltd., Shanghai, China; 4 Clinical Pharmacology, Development China, Pfizer Investment Co. Ltd., Beijing, China; 5 China Statistics, Global Biometrics & Data Management, Pfizer Inc., Shanghai, China; 6 Clinical Pharmacology, Development China, Pfizer Pharmaceutical Ltd., Shanghai, China

**Keywords:** Talazoparib, Pharmacokinetics, Safety, Efficacy, Chinese, Advanced solid tumor

## Abstract

Talazoparib, a poly(ADP-ribose) polymerase inhibitor, has demonstrated efficacy in the treatment of advanced breast and prostate cancers in Western populations. This open-label, phase 1 study investigated the pharmacokinetics, safety, and antitumor activity of talazoparib monotherapy in Chinese patients with advanced solid tumors. Molecularly unselected patients (≥18 years) with advanced solid tumors resistant to standard therapy received talazoparib (oral, 1 mg once daily). Primary endpoint was characterization of single-dose and steady-state pharmacokinetics. Secondary endpoints evaluated safety, unconfirmed objective response rate (ORR), and duration of response. The safety population comprised 15 Chinese patients (median [range] age 53.0 [31.0–72.0] years). Single-dose median time to first occurrence of maximum observed concentration was 1.9 h; concentrations then declined with a mean terminal half-life (t_1/2_) of 67 h. Following multiple dosing, median T_max_ was approximately 1.85 h with steady state generally achieved by Day 21. Treatment-related treatment-emergent adverse events (TEAEs) occurred in 86.7% (13/15) of patients (grade 3, 20.0%; grade 4, 13.3%). Two patients (13.3%) experienced serious treatment-related TEAEs. ORR (investigator-assessed) was 6.7% (95% CI: 0.2–31.9); one patient (6.7%) had a partial response. In patients with measurable disease at baseline, the ORR was 9.1% (1/11; 95% CI: 0.2–41.3; duration of response: 114 days); stable disease was achieved by 36.4% (4/11) of patients, and 54.5% (6/11) progressed by data cut-off. In Chinese patients with advanced solid tumors, the pharmacokinetic profile of talazoparib monotherapy (1 mg/day) was consistent with other patient populations. TEAEs were generally manageable with no unexpected safety findings. *(ClinicalTrials.gov: NCT04635631 [prospectively registered November 19, 2020])*

**Supplementary Information:**

The online version contains supplementary material available at 10.1007/s10637-023-01351-w.

## Introduction

In 2020, there were almost 10 million cancer deaths and approximately 19.3 million new cancer cases worldwide [1]. China alone accounts for 30% of global cancer-related deaths (about 3 million) and nearly 25% of new cancer cases (23.8%; approximately 4.6 million) in 2020. The most common causes of cancer in China include lung (17.9%), colorectal (12.2%), stomach (10.5%), breast (9.1 %), and liver (9%) cancer [2]. By 2040, the burden of cancer is expected to rise by 47% to 28.4 million cases globally [1], and in China, the number of new cancer cases is expected to reach 6.85 million [3]. This highlights the clear and unmet need for greater anticancer therapy options for Chinese patients.

Poly(ADP-ribose) polymerase (PARP) inhibitors target the enzymes PARP1 and PARP2, which play key roles in DNA repair [4, 5]. PARP inhibitors block the catalytic activity of PARP enzymes and some PARP inhibitors also trap PARP enzymes at sites of single-stranded DNA breaks. In cells with alterations in genes involved directly or indirectly with homologous recombination repair (HRR), such as *BRCA1/2*, this can lead to the irreparable accumulation of double-stranded DNA damage and cell death [4–7]. Talazoparib is both a PARP inhibitor and a particularly potent PARP trapper, a property that is associated with cytotoxicity in preclinical models [7, 8].

Talazoparib (oral, 1 mg once daily [QD]) is approved as a monotherapy for the treatment of patients with human epidermal growth factor receptor 2 (HER2)-negative advanced breast cancer with a germline *BRCA1/2* mutation in the US, EU, and other countries [9, 10]. The pharmacokinetic (PK) profile, safety, and efficacy of talazoparib has been established in Western populations for the treatment of breast and prostate cancer. The phase 1, first-in-human trial (NCT01286987) of talazoparib involved patients with advanced cancers that harbored germline *BRCA1/2* mutations or were predicted to be sensitive to PARP inhibition [11]. The recommended dose of talazoparib was identified as 1 mg QD, and the median time to maximum observed concentration (C_max_) was generally reached within 2 h. After daily dosing, steady-state plasma concentrations were reached by 2 weeks [11]. The safety profile of talazoparib has been characterized across several clinical studies; common adverse events (AEs) include hematologic AEs such as anemia, neutropenia, and thrombocytopenia, and nonhematologic AEs such as fatigue, nausea, headache, vomiting, alopecia, diarrhea, and decreased appetite [9, 12, 13]. In the phase 3 EMBRACA trial (NCT01945775) involving patients with advanced breast cancer and a germline *BRCA* alteration, talazoparib monotherapy provided a significant improvement in median progression-free survival versus standard chemotherapy (8.6 months vs. 5.6 months; hazard ratio for disease progression or death, 0.54; 95% confidence interval [CI]: 0.41–0.71; *P* < 0.001). Among all patients in the talazoparib arm, 1% were male, 67% were White, 11% were Asian, and 4% were Black or African American [14]. In the phase 2 TALAPRO-1 trial (NCT03148795), talazoparib monotherapy has also demonstrated efficacy in heavily pretreated men with metastatic castration-resistant prostate cancer and HRR alterations in Western populations (87% of men were white, 3% were Black, and 2% were Asian) [15].

When this study was initiated, no Chinese patients were involved in prior global talazoparib studies. The purpose of this open-label phase 1 study (NCT04635631) was to evaluate the PK, safety, and antitumor activity of talazoparib monotherapy in Chinese patients with advanced solid tumors.

## Methods

### Study design and patients

Patients (aged ≥18 years) had locally advanced or metastatic solid tumors resistant to standard therapy, or for which no standard therapy had been available, as well as a baseline Eastern Cooperative Oncology Group performance status (ECOG PS) of 0 or 1. To be included, all patients were required to have adequate bone marrow, renal, and liver function. Additional inclusion and exclusion criteria are listed in Supplementary Table 1.

Prior clinical studies have demonstrated the maximum tolerated dose of talazoparib monotherapy to be 1 mg QD in Western and Japanese patients [11, 16]. Based on the established safety profile and approval of talazoparib monotherapy administered at 1 mg QD by the US Food and Drug Administration, European Medicines Agency, and other global health authorities [9, 10], a 3+3 study design was not required.

A single dose of talazoparib 1 mg was administered orally on Day –9. From Cycle 1 Day 1, patients received talazoparib 1 mg QD orally on a continuous basis at approximately the same time each day (preferably in the morning) until disease progression, death, unacceptable toxicity, or withdrawal of consent. Dose interruptions or reductions were not required unless toxicity persisted at grade 2 for ≥7 days. Daily dosing was paused for grade ≥3 hematologic toxicities. Supportive care including blood products was allowed as appropriate per local guidance. To minimize any drug-drug interaction effects relating to talazoparib exposure and potential alteration to efficacy, co-medication with P-glycoprotein (P-gp) inhibitors and inducers was prohibited for Cycle 1 when intensive PK assessments were scheduled after both single-dose and multiple-dose talazoparib administrations. After Cycle 1, only potent P-gp inhibitors were prohibited to accommodate more co-medication options.

The final protocol and any amendments were reviewed and approved by the independent ethics committees at each of the investigational centers participating in the study. This study was conducted in compliance with the ethical principles originating in or derived from the Declaration of Helsinki and in compliance with all International Council for Harmonization Guidelines for Good Clinical Practice. Informed consent was obtained from all individual patients included in the study.

### Sample size consideration

To support registration of patients with metastatic castration-resistant prostate cancer (mCRPC) and other future potential indications in China, it was determined that 12 evaluable patients were needed to characterize the Chinese PK profile. This number was based on available global talazoparib PK data and with the intention to satisfy regulatory requirements by the China National Medical Products Administration for PK evaluation in a Chinese population. Considering there will be non-evaluable patients, it was estimated that approximately 15 patients were needed to be enrolled.

### Endpoints

The primary endpoint was to characterize the single-dose and steady-state PK of talazoparib. Key secondary endpoints included evaluation of the incidence and severity of AEs and efficacy, as evaluated by unconfirmed objective response rate and duration of response.

### Pharmacokinetic assessments

To assess the single-dose PK profile, patients took talazoparib on Day –9 of a 9-day (216 h) lead-in period; blood samples were collected at pre-dose and at 0.5, 1, 2, 4, 8, 24, 48, 96, 168, and 216 h post-dose. For the steady-state PK profile, serial samples after multiple doses of talazoparib were collected on Day 22 of the first cycle at pre-dose and at 0.5, 1, 2, 4, 8, and 24 h post-dose. Prior to serial PK sampling (Cycle 1 Day –9, Cycle 1 Day 22), patients were required to fast for at least 8 h before dosing and they had to continue fasting for 2 h after dosing to control the variability due to food effect on C_max_. For the other PK sampling points, talazoparib could have been taken with or without food. On clinic visit days, talazoparib was administered after completion of a pre-dose blood sampling for PK and assessments. Blood samples of approximately 3 mL, to provide a minimum of 1.5 mL plasma, for measurement of talazoparib concentrations were collected.

### Safety assessments

AE reporting included data up to 28 days after last dose of study drug or to start of new anticancer drug therapy Day -1 (whichever came first). For AEs, Medical Dictionary for Regulatory Activities (MedDRA), version 24.0, coding was applied.

### Efficacy assessments

Tumor assessments were performed on Day 29 and every 8 weeks thereafter for the initial 12 cycles, regardless of any dose interruptions or dose delays, and then performed per local standard practice after completion of Cycle 12. Tumor assessments were repeated at the end of study visit if more than 6 weeks had passed since the last evaluation. Unconfirmed objective response by investigator assessment was defined as a complete (disappearance of all target lesions) or partial (at least 30% decrease in the sum of diameters of target lesions) response recorded from Cycle 1 Day 1 until disease progression, start of subsequent anticancer therapy, or death due to any cause. Given the exploratory nature of the efficacy endpoint, confirmation of response was not required. Duration of response, calculated as the time from first documentation of complete response or partial response to date of first documentation of objective progression or death, was only calculated for the subgroup of patients with an unconfirmed objective response.

### Statistical analyses

All patients who had received at least 1 dose of talazoparib were included in the safety and efficacy analysis sets. All treated patients with at least 1 PK concentration in the single-dose and/or multiple-dose PK part were included in the PK concentration analysis set, and all treated patients with at least 1 of the PK parameters of primary interest in the single-dose and/or multiple-dose PK part were included in the PK parameter analysis set. All patients in the PK parameter analysis set who completed both the single-dose PK and multiple-dose PK parts without major protocol deviations were included in the PK evaluable analysis set.

Talazoparib PK parameters were calculated for each patient and each treatment, as applicable, using non-compartmental analysis of concentration-time data. Summary statistics were calculated by setting concentration values below the lower limit of quantification to zero.

## Results

### Patients and disposition

A total of 18 Chinese patients were screened, and 15 were enrolled and treated with talazoparib. At data cut-off (August 8, 2021), 40.0% (6/15) remained on study treatment and 60% (9/15) discontinued, primarily due to progressive disease (46.7% [7/15]). Among the 9 patients who discontinued, 2 declined to enter follow-up. Of the 7 patients who entered follow-up, 40.0% (6/15) completed the follow-up and 6.7% (1/15) were still ongoing.

The safety population comprised 15 patients (median [range] age 53.0 [31.0–72.0] years) who were enrolled and treated with ≥1 dose of talazoparib (Table 1). Disease characteristics are also summarized in Table 1. Most patients had a primary diagnosis of breast cancer (60% [9/15]) or ovarian cancer (26.7% [4/15]). Twelve (80.0%) patients completed both the single- and multiple-dose PK parts and were included in the PK evaluable analysis population.

**Table 1:** Baseline patient and disease characteristics

	**Safety population** **(*N*=15)**
Age, years	
18 to <45, *n* (%)	4 (26.7)
45 to <65, *n* (%)	8 (53.3)
≥65, *n* (%)	3 (20.0)
Median (range)	53.0 (31.0–72.0)
Gender, *n* (%)	
Female	15 (100.0)
Racial designation, *n* (%)	
Chinese	15 (100.0)
BMI (kg/m^2^), median (range)	23.4 (19.9–29.8)
ECOG performance status, *n* (%)	
0	7 (46.7)
1	8 (53.3)
Primary diagnosis	
Breast cancer, *n* (%)	9 (60.0)
Duration since diagnosis (months), median (range)	78.3 (24.3–155.9)
Ovarian cancer, *n* (%)	4 (26.7)
Duration since diagnosis (months), median (range)	21.9 (8.2–0.9)
Fallopian tube cancer, *n* (%)	1 (6.7)
Duration since diagnosis (months), median (range)	11.8 (11.8–11.8)

BMI (kg/m^2^) = weight (kg) / [height (cm) × 0.01]^2^. Baseline is defined as the latest non-missing value on or prior to the date of the first dose of study treatment

*BMI* body mass index, *ECOG* Eastern Cooperative Oncology Group

All 15 patients included in the safety population received at least 1 prior anticancer drug therapy. Most (66.7% [10/15]) patients had received ≥4 regimens of prior anticancer drug therapy. The most frequently received prior drug treatments were paclitaxel (80% [12/15]) and docetaxel (60% [9/15]), followed by capecitabine, carboplatin, cisplatin, and cyclophosphamide (53.3% [8/15] patients each).

#### Pharmacokinetics

**Fig. 1:**
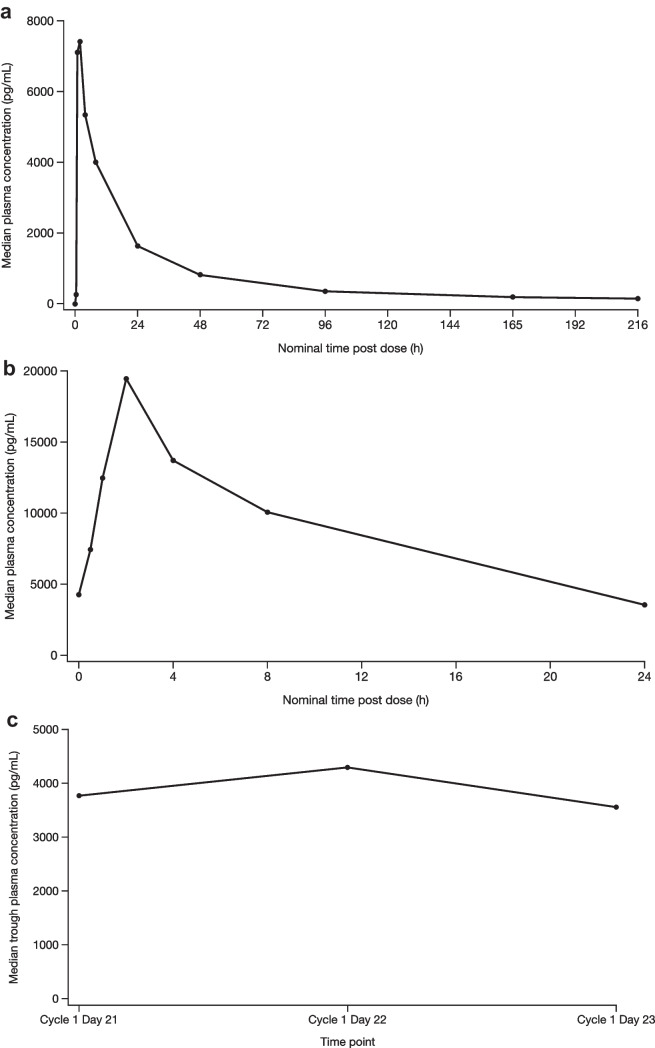
^a^PK concentration population. ^b^One patient was excluded as PK were potentially impacted by an adverse event. Single dose: Pre-dose and 0.5, 1, 2, 4, 8, 24, 48, 96, 168, and 216 h post-dose on Day –9. The lower limit of quantificationis 25.0 pg/mL. Multiple dose: Pre-dose and 0.5, 1, 2, 4, 8, and 24 h post-dose on Cycle 1 Day 22. The lower limit of quantification is 25.0 pg/mL. Summary statistics were calculated by setting concentration values below the lower limit of quantification to zero h hour, PK pharmacokinetics. Linear median plasma talazoparib concentration-time profile following (**a**) single oral lead-in dose,^a^ (**b**) multiple oral doses (Day 22 steady state),^a,b^ and (**c**) median plasma talazoparib trough (pre-dose) concentrations by day^a,b^

Median plasma talazoparib concentration-time linear profiles following single oral dose (lead-in period) and multiple oral doses (Day 22; steady state) are presented in Fig. 1a and Fig. 1b, respectively. The semi-log profiles are shown in Supplementary Fig. 1a–b. A summary of PK parameters is presented in Table 2. Median trough (pre-dose) concentrations by day are presented in Fig. 1c. For the multiple-dose analyses, 1 patient was excluded since PK were potentially impacted by an AE. Following a single oral dose, talazoparib was absorbed rapidly. The single-dose median T_max_, the time to first occurrence of C_max_, was 1.90 h. After attainment of C_max_, concentrations declined with a mean t_1/2_ of 67 h (Fig. 1a and Supplementary Fig. 1a). Mean apparent oral clearance was 4.798 L/h and mean volume of distribution was 456.8 L (Table 2). Following multiple oral dosing on Day 22, median T_max_ was approximately 1.85 h. Based on the area under the plasma concentration-time profile from time zero to time tau (AUC_tau_) calculation, mean apparent oral clearance was 5.190 L/h. Steady state was generally achieved by Day 21 based on similar median trough (pre-dose) concentrations (Fig. 1c).

The observed accumulation ratio for AUC_tau_ was 2.286. The geometric mean of the predicted accumulation ratio R_ss_ was 1.072, which compared AUC_tau_ for multiple-dose administration to AUC_inf_ for single-dose administration to assess the linearity in PK exposure from single dose to steady state. Between-patient variability in plasma talazoparib exposure following single-dose (lead-in) and multiple-dose (Day 22) administration based on geometric %CV (coefficient of variation) that ranged from 41% to 32% for C_max_ and from 29% to 32% for AUC (from time zero to infinity [AUC_inf_], from time zero to the time of the last quantifiable concentration [AUC_last_], and AUC_tau_).

**Table 2:** Descriptive summary of plasma talazoparib PK

**Parameters**^**a**^	**PK parameter analysis population (*N*=15)**
Single dose	
N2, N3	15, 10
AUC_inf_, ng.h/mL	208.3 (31)
AUC_last_, ng.h/mL	172.0 (32)
AUC_tau_, ng.h/mL	86.54 (29)
CL/F, L/h	4.798 (31)
C_max_, ng/mL	8.506 (41)
t_1/2_, h	67.00 ± 11.779
T_max_, h	1.90 (0.517–7.63)
V_Z_/F, L	456.8 (37)
Multiple dose^b^	
N2, N3	11, 7
AUC_tau_, ng.h/mL	192.9 (29)
CL/F, L/h	5.190 (29)
C_max_, ng/mL	19.85 (32)
C_min_, ng/mL	3.204 (43)
R_ac_	2.286 (22)
R_ss_	1.072 (24)
T_max_, h	1.85 (0.533–4.27)

^a^Data are geometric means (geometric %CV), except median (range) for T_max_ and arithmetic mean ± standard deviation for t_1/2_

^b^One patient was excluded as PK were potentially impacted by an adverse event

*AUC*_*inf*_ area under the plasma concentration-time profile from time zero to infinity, *AUC*_*last*_ area under the plasma concentration-time profile from time zero to the time of the last quantifiable concentration, *AUC*_*tau*_ area under the plasma concentration-time profile from time zero to time tau (τ), *CL/F* apparent clearance, *C*_*max*_ maximum observed concentration, *C*_*min*_ minimum plasma concentration, *CV* coefficient of variation, *h* hour, *N2* number of patients contributing to summary statistics, *N3* number of patients contributing to summary statistics for AUC_inf*,*_, *CL/F* t_1/2_ and V_Z_/F (single dose) and for R_ss_ (multiple dose), *PK* pharmacokinetics, *R*_*ac*_ observed accumulation ratio, *R*_*ss*_ steady-state accumulation ratio, *T*_*max*_ time to first occurrence of *C*_*max*_, *t*_*1/2*_ terminal half-life, *V*_*Z*_*/F* apparent volume of distribution

### Safety

The median duration of treatment with talazoparib was 2.17 (range: 0.82–7.98) months and the median relative dose intensity was 76.95% (range: 39.90%–96.71%). Of all patients, 93.3% (14/15) and 86.7% (13/15) experienced all-causality or treatment-related treatment-emergent adverse events (TEAEs), respectively (Table 3).

**Table 3:** All-causality and treatment-related treatment-emergent adverse events

	**Safety population** **(*N*=15)**	
	All-causality *n* (%)	Treatment-related *n* (%)
Any AE	14 (93.3)	13 (86.7)
Grade 3 or 4 AE^a^	9 (60.0)	5 (33.3)
Gamma-glutamyltransferase increased	4 (26.7%)	0
Anemia	3 (20.0)	3 (20.0)
Neutrophil count decreased	3 (20.0)	3 (20.0)
Platelet count decreased	3 (20.0)	3 (20.0)
Grade 5 AE (Death due to AE)	0	0
Patients with any SAE^b^	2 (13.3)	2 (13.3)
Anemia	1 (6.7)	1 (6.7)
Hypernatremia	1 (6.7)	0
Hyponatremia	1 (6.7)	1 (6.7)
Neutrophil count decreased	1 (6.7)	1 (6.7)
Platelet count decreased	1 (6.7)	1 (6.7)
AEs leading to dose interruption	3 (20.0)	2 (13.3)
AEs leading to dose reduction	3 (20.0)	3 (20.0)
AEs leading to discontinuation	1 (6.7)	1 (6.7)

^a^Occurring in ≥20% of patients

^b^One patient experienced treatment-related SAEs of thrombocytopenia, neutropenia, and anemia. The second patient experienced a treatment-related SAE of hyponatremia

MedDRA v24.0 coding dictionary was applied. SAEs were based on the investigator's assessment

Severity counts are based on the maximum severity or grade of events. When the action taken is both dose reduction and dose interruption, dose reduction is checked as the most severe action

*AE* adverse event, *MedDRA* Medical Dictionary for Regulatory Activities, *SAE* serious adverse event

All-causality and treatment-related AEs led to dose interruptions in 20.0% (3/15) and 13.3% (2/15) of patients, respectively. Twenty percent (3/15) of patients experienced 1 or more treatment-related dose reduction due to treatment-related AEs, including anemia, decreased neutrophil count, decreased platelet count, and neutropenia (Table 3). One (6.7%) patient permanently discontinued because of treatment-related decreased neutrophil count. Prior to discontinuation, this patient experienced dose reductions from 1 mg to 0.5 mg and then to 0.25 mg due to grade 3 and 4 AEs. Further reduction from 0.25 mg was not allowed according to the protocol.

The most common (≥20%) any-grade treatment-related TEAEs were anemia (46.7% [7/15]), decreased neutrophil count (46.7% [7/15]), decreased white blood cell count (46.7% [7/15]), elevated alanine aminotransferase (33.3% [5/15]), elevated aspartate aminotransferase (33.3% [5/15]), decreased lymphocyte count (33.3% [5/15]), and decreased platelet count (33.3% [5/15]). The incidence of grade 3 and 4 treatment-related TEAEs is summarized in Table 3. Grade 3 or 4 treatment-related AEs were experienced by 20% and 13.3% of patients, respectively. No grade 5 all-causality or treatment-related TEAEs were reported. The most common total treatment-related grade 3 or 4 TEAEs were anemia (20.0% [3/15]; no grade 4), decreased neutrophil count (20.0% [3/15]), decreased platelet count (20.0% [3/15]), hyponatremia (6.7% [1/15]; no grade 4), and neutropenia (6.7% [1/15]; no grade 4; Table 3). Serious all-causality and treatment-related TEAEs were observed in 2 patients (13.3%) and are summarized in Table 3.

### Efficacy

Of the 15 treated patients, the unconfirmed objective response rate based on investigator assessment was 6.7% (95% CI: 0.2–31.9, Table 4). One patient (6.7%) had a partial response, (Table 4), 26.7% (4/15) of patients achieved stable disease, and 6.7% (1/15) were categorized with non-complete response/non-progressive disease. Disease control was observed in 6 patients (40.0% [95% CI: 16.3–67.7], Table 4) based on best overall response.

Of the 11 patients with measurable disease at baseline, one (9.1%) had a best overall response of partial response and remained as partial response at data cut-off (95% CI: 0.2–41.3; duration of response: 114 days). For patients with measurable disease at baseline, stable disease was achieved in 36.4% (4/11) of patients and 54.5% (6/11) had progressed by the data cut-off (Table 4).

**Table 4:** Best overall response and unconfirmed objective response based on investigator assessment

	**Efficacy analysis** **set** **(*N*=15)**	**Patients with measurable disease** **(*N*=11)**
Confirmed or unconfirmed best overall response, *n* (%)		
Complete response	0	0
Partial response	1 (6.7)	1 (9.1)
Stable disease	4 (26.7)	4 (36.4)
Non-complete response/non-progressive disease	1 (6.7)	0
Progressive disease	7 (46.7)	6 (54.5)
Not evaluable^a^	2 (13.3)	0
Objective response,^b^ *n* (%, [95% CI]^c^)	1 (6.7 [0.2–31.9])	1 (9.1 [0.2–41.3]
Disease control,^d^ *n* (%, [95% CI]^b^)	6 (40.0 [16.3–67.7])	5 (45.5 [16.7–76.6])

^a^Reason for not evaluable include no post-baseline assessments due to other reasons (1/15 [6.7%]) and occurrence of stable disease less than 6 weeks after initiation of treatment (1/15 [6.7%])

^b^Objective response included complete and partial responses

^c^Clopper-Pearson method used

^d^Disease control includes complete and partial responses, stable disease, and non-complete response/non-progressive disease

*CI* confidence interval

## Discussion

In this phase 1 study of Chinese patients with advanced solid tumors, talazoparib was absorbed rapidly and steady state was generally reached by Day 21, which is consistent with the PK profile previously established in Western and Asian patients (Supplementary Table 2). In the first phase I study of talazoparib monotherapy (0.025–1.1 mg QD) in a Western population of 110 patients with advanced solid tumors, de Bono et al demonstrated rapid absorption of talazoparib, with C_max_ that was generally reached within 2 h and a steady state achieved approximately 2 weeks after initiation of daily dosing [11]. Plasma elimination of talazoparib followed biphasic kinetics with a long t_1/2_ of approximately 2 days, and mean accumulation ratio was 2.4-fold at steady state. Plasma concentrations, C_max_, and area under the plasma concentration-time curve (AUC) estimates increased approximately with doses ranging from 0.025 to 1.1 mg following multiple daily dosing [11].

In a recent 2-part phase 1 study of talazoparib monotherapy in 9 Japanese patients with locally advanced or metastatic solid tumors, Naito et al reported that the single dose C_max_ (13.78 ng/mL) was reached at 0.97 (0.5–2.0) h and the multiple dose C_max_ (32.84 ng/mL) was reached 1.03 (0.7–1.9) h in part 1 of the study [16]. In part 2 of the follow-up study involving 19 Japanese patients with germline *BRCA*-mutated advanced breast cancer, Kotani et al reported PK data that were consistent with the part 1 results and with observations from the EMBRACA and ABRAZO trials [16–18]. The geometric mean talazoparib C_trough_, calculated using steady-state trough concentrations at each visit for each patient, was determined to be 3346 pg/mL [18], which is similar to that observed in this phase 1 study (Fig. 1C).

In addition to the above-mentioned cross study comparison, a modeling approach also supported comparable PK profiles among Asian and Western populations. In an analysis from 2020, Yu et al developed a population PK model based on the data from four clinical trials investigating talazoparib monotherapy in patients with advanced cancers. The analysis included two phase 1 trials (PRP-001 and PRP-002), the phase 2 ABRAZO trial, and the phase 3 EMBRACA trial. Patients had both solid (PRP-001, ABRAZO, and EMBRACA) and hematologic (PRP-002) malignancies [17]. The PK population dataset included 490 patients, of which 73.7% (361/490) were White, 3.3% (16/490) were Black, 8.4% (41/490) were Asian, 1.8% (9/490) were another ethnicity, and 12.9% (63/490) did not have a reported ethnicity. The authors found that talazoparib PK was well characterized by a 2-compartment model with first-order absorption and absorption lag time. The reported mean apparent oral clearance and apparent volume of distribution of the central compartment were estimated at 6.36 L/h (27.0% interpatient variability) and 162 L (4.79% interpatient variability), respectively. Based on covariate analysis, no dose adjustment for talazoparib was required based on a patient’s age, sex, baseline body weight, or Asian race [17]. PK profiles demonstrated here in our phase 1 study are comparable to other Asian/Western populations observed in clinical trials or using a modeling approach.

We report that TEAEs with talazoparib monotherapy were generally manageable in Chinese patients with no unexpected safety findings. Most (86.7%) patients experienced treatment-related TEAEs, the majority of which were grade 1–2. One-third of patients experienced grade 3–4 treatment-related AEs and no grade 5 TEAEs were reported. This is consistent with observations by Kotani et al in Japanese patients with solid tumors treated with talazoparib monotherapy, in which all patients experienced treatment-related AEs, 52.6% (10/19) of patients experienced grade 3–4 treatment-related AEs, and there were no grade 5 AEs [18].

The most common total grade 3–4 AEs in our study were hematologic, including treatment-related anemia (20% [3/15]), decreased neutrophil count (20% [3/15]), and decreased platelet count (20% [3/15]). Hematologic AEs are a known class effect of PARP inhibitors, and similar to results seen in Asian and Western patient populations [12, 13, 19, 20]. Kotani et al also reported that anemia was the most common treatment-related AE, occurring in 68.4% (13/19) of Japanese patients [18]. This observation is consistent with data from the TALAPRO-1 and EMBRACA trials, which evaluated talazoparib monotherapy in patients with mCRPC and advanced breast cancer, respectively [13–15]. The most common grade 3–4 TEAEs in TALAPRO-1 were anemia (31% [39/127]), thrombocytopenia (9% [11/127]), and neutropenia (8% [10/127]) [15]. In EMBRACA, the most common grade 3–4 TEAEs were anemia (39% [112/286]), neutropenia (21% [60/286]), and thrombocytopenia (15% [42/286]) [14].

Our study involved molecularly unselected Chinese patients with advanced solid tumors. The disease control rate was 45.5% (95% CI: 16.7–76.6). This is consistent with findings reported from the first part of the phase 1 study reported by Naito et al of talazoparib monotherapy in a population of molecularly unselected Japanese patients with solid tumors. In the Naito et al study, the overall disease control rate was 44.4% and included 2 patients with stable disease [16].

In conclusion, talazoparib monotherapy in Chinese patients with advanced solid tumors showed no new safety signals. The PK profile was similar to reports from other non-Chinese populations supporting the established 1 mg QD dosing. Our findings support the conclusion that the safety and PK profiles of talazoparib are consistent between populations. This study also provides supportive data for the continued evaluation of talazoparib combined with enzalutamide in Chinese patients in global clinical trials, including TALAPRO-2 (mCRPC unselected for HRR alterations; NCT03395197) and TALAPRO-3 (castration-sensitive prostate cancer with HRR alterations; NCT04821622) [21–23].

## Supplementary Information

Below is the link to the electronic supplementary material.

Supplementary file1 (PDF 306 KB)

## Data Availability

Upon request, and subject to review, Pfizer will provide the data that support the findings of this study. Subject to certain criteria, conditions and exceptions, Pfizer may also provide access to the related individual de-identified participant data. See https://www.pfizer.com/science/clinical-trials/trial-data-and-results for more information.
