# Supplementary material for: Pharmacokinetics, safety, and antitumor activity of talazoparib monotherapy in Chinese patients with advanced solid tumors
Source: Invest New Drugs. 2023 May 12;41(3):503–11. doi: 10.1007/s10637-023-01351-w (PMC10290043; doi:10.1007/s10637-023-01351-w)
Supplement: Supplementary file 1 — Supplementary file1 (PDF 306 KB) [file 10637_2023_1351_MOESM1_ESM.pdf]

Supplementary Materials

Pharmacokinetics, safety, and antitumor activity of talazoparib monotherapy in Chinese patients with advanced solid tumors

Yang Luo,[1#] Ying Cheng,[2#] Chunjiao Wu,[2] Hui Ye,[3] Naihan Chen,[4] Fan Zhang,[5] Hua Wei,[6] Binghe Xu[1*]

# Represents co-first author; * Represents corresponding author

[1]National Cancer Center/Cancer Hospital, Chinese Academy of Medical Sciences and Peking Union Medical College, Beijing, China; [2]Department of Medical Oncology, Jilin Cancer Hospital, Changchun, Jilin, China; [3]Clinical Development, Development China, Pfizer Pharmaceutical Ltd., Shanghai, China; [4]Clinical Pharmacology, Development China, Pfizer Investment Co. Ltd., Beijing, China; [5]China Statistics, Global Biometrics & Data Management, Pfizer Inc., Shanghai, China; [6]Clinical Pharmacology, Development China, Pfizer Pharmaceutical Ltd., Shanghai, China

Corresponding author and contact details:

Binghe Xu

Email: xubinghe@medmail.com.cn

Target Journal: *Investigational New Drugs*

Supplementary Table 1 Inclusion and exclusion criteria

Inclusion criteria
Patient and disease characteristics
• Male or female patients aged ≥18 years[a]
• Histological or cytological diagnosis of locally advanced or metastatic solid tumor resistant to standard therapy or for which no standard therapy had been available
• ECOG PS 0 or 1
• Adequate bone marrow,[b] renal,[c] and liver[d] function
• Resolution of acute effects of any prior therapy to baseline severity or CTCAE grade ≤1, except for adverse events not constituting a safety risk by investigator judgment
**Exclusion criteria**
Medical conditions
• Brain metastases
• Previous high-dose chemotherapy requiring stem cell rescue
• Myocardial infarction ≤6 months before starting therapy, symptomatic congestive heart failure (New York Heart Association class III or IV), unstable angina, or unstable cardiac arrhythmia requiring medication[e]
• Hypertension that could not be controlled by medications (>150/100 mm Hg despite optimal medical therapy)
• Known or suspected hypersensitivity to active ingredients/excipients
Prior or concomitant therapy
• Major surgery ≤4 weeks prior to the first dose of study treatment
• Radiation therapy ≤4 weeks prior to the first dose of study treatment or palliative radiotherapy for the treatment of painful bony lesions ≤2 weeks prior to the first dose of study treatment
• Any antitumor systemic cytotoxic therapies ≤4 weeks prior to the first dose of study treatment (6 weeks for nitrosoureas or mitomycin-C), treatment with immune modulators (including, but not limited to, corticosteroids [at a prednisone-equivalent dose of >10 mg/day], cyclosporine, and tacrolimus[f])
• Prior irradiation to >25% of the bone marrow
• Current or anticipated use of P-gp inhibitor and/or inducer within 7 days prior to study intervention from lead-in to end of Cycle 1; concomitant use of potent P-gp inhibitor after Cycle 1 until the end of treatment
• Prior treatment with a PARP inhibitor

[a]Female patients were eligible to participate if they were not pregnant or breastfeeding. Patients of childbearing potential must have been willing to use a highly effective contraceptive method during the intervention period and for at least 7 months after the last dose of study intervention

[b]ANC ≥1500/mm^3 or ≥1.5×10^9/L without the use of growth factor ≤14 days before obtaining the hematology laboratory tests; platelets ≥100,000/mm^3 or ≥100×10^9/L without the use of platelet transfusions or growth factors within 14 days before obtaining the hematology laboratory tests; hemoglobin ≥9 g/dL, with last transfusion at least 14 days prior to the hematology laboratory tests

[c]Estimated CLcr ≥60 mL/min as calculated using the Cockcroft-Gault Formula

[d]Serum TBili ≤1.5×ULN unless the patient had documented Gilbert syndrome (≤3×ULN for Gilbert syndrome); AST and ALT ≤2.5×ULN (≤5.0×ULN if there was liver involvement by the tumor); alkaline phosphatase ≤2.5×ULN (≤5×ULN in case of bone metastasis)

[e]Stable cardiac arrhythmia (eg, chronic atrial fibrillation controlled by medication) could be eligible

[f]Locally active treatments (such as Beconase) were allowed within 4 weeks prior to the first dose of study treatment

ANC, absolute neutrophil count; ALT, alanine aminotransferase; AST, aspartate aminotransferase; CLcr, creatinine clearance; CTCAE, Common Terminology Criteria for Adverse Events; ECOG PS, Eastern Cooperative Oncology Group performance status; PARP, poly(adenosine diphosphate-ribose) polymerase; P-gp, P-glycoprotein; TBili, total bilirubin; ULN, upper limit of normal

Supplementary Table 2 Summary of PK parameters from different study populations

	**This study NCT04635631 *N*=15 (single dose) *N*=14[a] (multiple dose)**	**Naito et al 2021 [1] NCT03343054 *N*=6**	**de Bono et al 2017 [2] NCT01286987 *N*=5 (single dose) *N*=6 (multiple dose)**
Dosing	1 mg QD orally	1 mg QD orally	1 mg QD orally
Population	100% Chinese	100% Japanese	Enrolled at 5 sites in the US and 1 in the UK
Parameters[b]			
Single dosing			
C_{max}, ng/mL	8.506 (41)	13.78 (26)	10.6 (4.22)
T_{max}, h	1.90 (0.517–7.63)	0.97 (0.5–2.0)	1.03 (0.73–2.07)
AUC_{last}, ng.h/mL	172.0 (32)	-	-
AUC_{tau}, ng.h/mL	86.54 (29)	-	182.00 (62.40)
CL/F, L/h	4.798 (31)	-	5.39 (1.59)
V_Z/F, L	456.8 (37)		415 (170)
$t_{1/2}$, h	67.00 ± 11.779	50.73 ± 10.1	52.9 ± 13.4
AUC_{inf}, ng.h/mL	208.3 (31)	199.7 (9)	-
Multiple dosing			
C_{max}, ng/mL	19.85 (32)	32.84 (14)	21.0 (7.99)
T_{max}, h	1.85 (0.533–4.27)	1.03 (0.7–1.9)	1.02 (0.75–2.00)
AUC_{tau}, ng.h/mL	192.9 (29)	244.7 (21)	-
CL/F, L/h	5.190 (29)	-	5.24 (1.39)
R_{ac}	2.286 (22)	2.32 (1.70–8.28)	-

[a]One patient was excluded from the multiple dosing analysis as PK were potentially impacted by an adverse event

[b]Data are geometric means (geometric %CV), except median (range) for T_{max}, arithmetic mean ± standard deviation for $t_{1/2}$, and median (range) for R_{ac} in Naito et al 2021.

AUC_{last}, area under the plasma concentration-time profile from time zero to the time of the last quantifiable concentration; AUC_{tau}, area under the plasma concentration-time profile from time zero to time tau (τ); CL/F, apparent clearance; C_{max}, maximum observed concentration; CV, coefficient of variation; h, hour; PK, pharmacokinetics; QD, once daily; R_{ac}, observed accumulation ratio; T_{max}, time to first occurrence of C_{max}; $t_{1/2}$, terminal half-life; V_Z/F, apparent volume of distribution

Supplementary Fig. 1 Semi-log median plasma talazoparib concentration-time profile following (a) single oral dose (lead-in)[a] and (b) multiple oral doses (Day 22 steady state)[a,b]

a

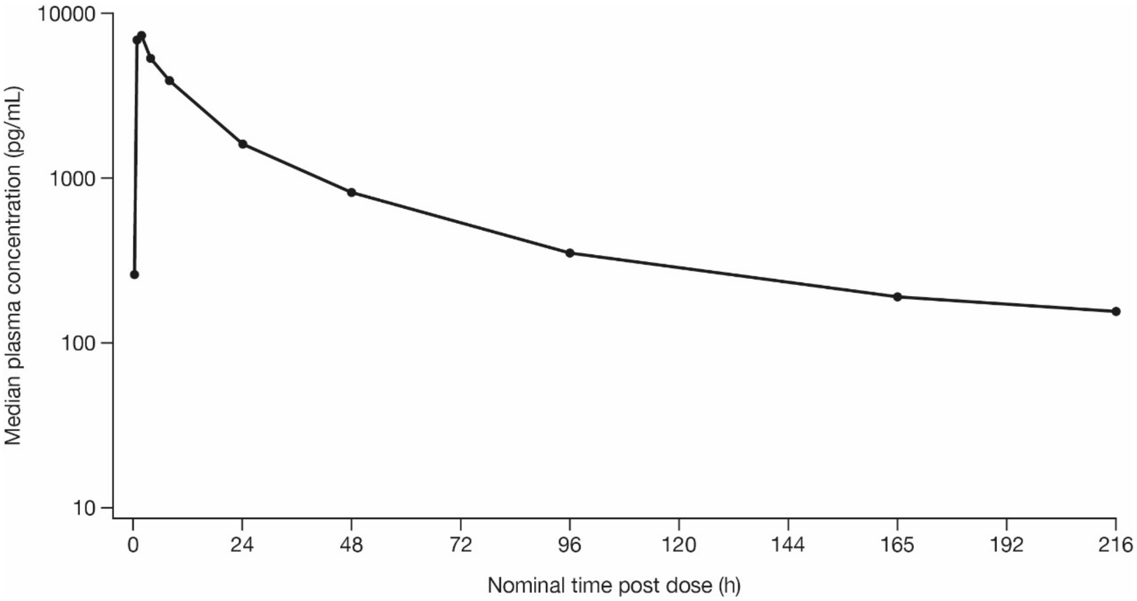

b

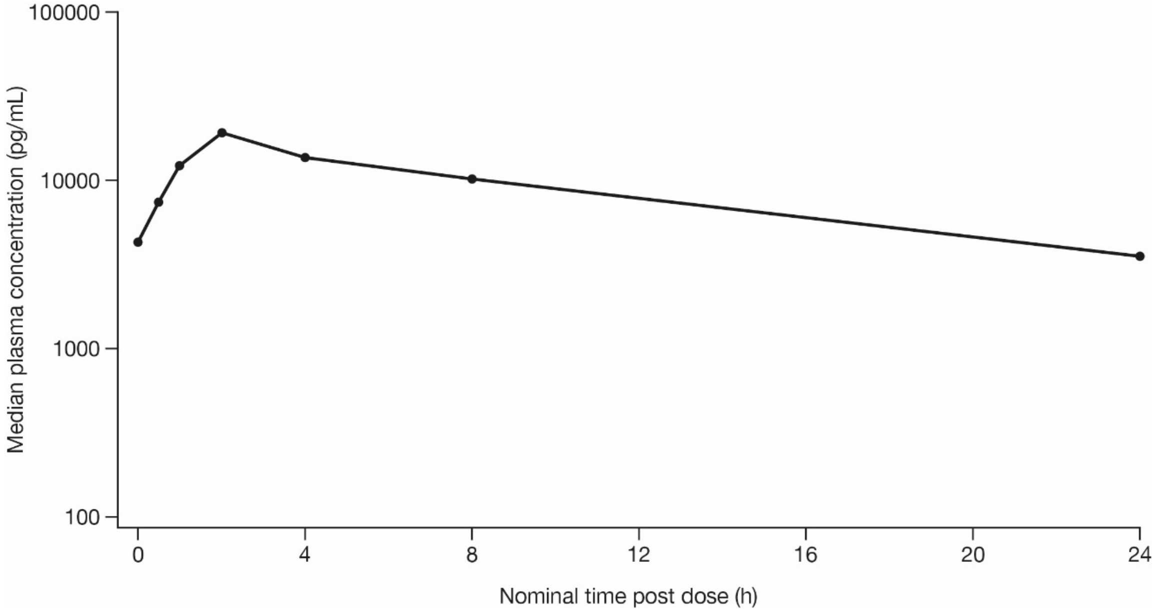

[a]PK concentration population

[b]One patient was excluded as PK were potentially impacted by adverse event

Single dose: Pre-dose and 0.5, 1, 2, 4, 8, 24, 48, 96, 168, and 216 h post-dose on Day –9. The lower limit of quantification is 25.0 pg/mL

Multiple dose: Pre-dose and 0.5, 1, 2, 4, 8, and 24 h post-dose on Cycle 1 Day 22. The lower limit of quantification is 25.0 pg/mL. Summary statistics have been calculated by setting concentration values below the lower limit of quantification to zero

h, hour; PK, pharmacokinetics

References

1. Naito Y, Kuboki Y, Ikeda M, Harano K, Matsubara N, Toyoizumi S, Mori Y, Hori N, Nagasawa T, Kogawa T (2021) Safety, pharmacokinetics, and preliminary efficacy of the PARP inhibitor talazoparib in Japanese patients with advanced solid tumors: phase 1 study. Invest New Drugs 39:1568-1576. https://doi.org/10.1007/s10637-021-01120-7
2. de Bono J, Ramanathan RK, Mina L, Chugh R, Glaspy J, Rafii S, Kaye S, Sachdev J, Heymach J, Smith DC, Henshaw JW, Herriott A, Patterson M, Curtin NJ, Byers LA, Wainberg ZA (2017) Phase I, dose-escalation, two-part trial of the PARP inhibitor talazoparib in patients with advanced germline *BRCA1/2* mutations and selected sporadic cancers. Cancer Discov 7:620-629. https://doi.org/10.1158/2159-8290.Cd-16-1250
